# *Salmonella* Durban meningitis: case report and genomics study

**DOI:** 10.1186/s12879-023-08308-7

**Published:** 2023-05-20

**Authors:** Christelle Nanga Diasi, Pieter-Jan Ceyssens, Alexandra Vodolazkaia, Marina Mukovnikova, Sarah Dorval, Olivia Bauraind, Wesley Mattheus

**Affiliations:** 1 Department of Pediatrics, CHC, Clinique MontLégia, Liège, Belgium; 2 University of Liège (ULiège), 5Th Master in Pediatrics, Liège, Belgium; 3 Bacterial Diseases Division, Communicable and Infectious Diseases, National Reference Centre for Salmonella and Shigella, Scientific Institute of Public Health, Wytsmanstreet 14, B-1050 Brussels, Belgium; 4 National Reference Centre for Salmonella and Shigella, Laboratory of Medical Microbiology, Communicable and Infectious Diseases, Scientific Institute of Public Health, Wytsmanstreet 14, B-1050 Brussels, Belgium; 5 Pediatric Infectious Disease, Clinique MontLégia,, CHC, Liège, Belgium; 6 Department of Pediatric Gastroenterology, Clinique MontLégia, CHC, Liège, Belgium

**Keywords:** Meningitis, Non-typhoid *Salmonella*, Paediatrics, Bacterial infection, *Salmonella* Durban

## Abstract

**Background:**

Bacterial meningitis caused by non-typhoid *Salmonella* can be a fatal condition which is more common in low and middle-income countries.

**Case presentation:**

We report the case of a *Salmonella* meningitis in a Belgian six-month old male infant. The first clinical examination was reassuring, but after a few hours, his general state deteriorated. A blood test and a lumbar puncture were therefore performed. The cerebrospinal fluid analysis was compatible with a bacterial meningitis which was later identified by the NRC (National Reference Center) as *Salmonella enterica* serovar Durban.

**Conclusions:**

In this paper, we present the clinical presentation, genomic typing, and probable sources of infection for an unusually rare serovar of *Salmonella*. Through an extended genomic analysis, we established its relationship to historical cases with links to Guinea.

## Background

*Salmonella*e are gram-negative bacilli belonging to the *Enterobacteriaceae* family. The most recent classification of *Salmonella* includes two species: *Salmonella enterica* and *Salmonella bongori* of which over 2500 different serovars have been identified based on somatic antigenic factors O, flagellar H and capsular Vi as explained by the World Health Organization (WHO) Collaborating Centre for Reference and Research on *Salmonella* [1].

Salmonellosis is a global public health problem; it is an infection acquired orally, causing usually a mild gastrointestinal infection, and which rarely requires an antibiotic treatment [2]. However, between 1 to 5.7% of patients may develop bacteremia, which is mostly benign, although osteoarticular or meningeal secondary involvement may occur [3]. Invasive infection occurs due to the distortion of local enteric immunity and is particularly seen in the young and the elderly or in individuals with predisposing conditions, particularly in immunosuppressed patients.

In high-income countries, *Salmonella* meningitis represents less than 1% of cases of bacterial meningitis confirmed in infants and children [4]. In contrast, the incidence reported in low and middle-income countries can reach up to 13% [5]. It has very rapid clinical deterioration and clinical manifestations which cannot be distinguished from any other bacterial meningitis. It is associated with a high incidence of complications, neurological disorders, high mortality, and a high percentage of relapses [6]. The prognosis is poor, especially, due to complications inherent to the infection, so early diagnosis is crucial to achieve a favorable outcome.

In Belgium, the surveillance of human salmonellosis relies on the voluntary submission of human *Salmonella* isolates to the National Reference Center (NRC). This center facilitates national epidemiological monitoring of human *Salmonella* infections, identifying outbreaks and tracking long-term spatial and temporal trends. This paper aims to firstly present the clinical case of an infant with recurrent Salmonellosis and meningitis caused by a *Salmonella* serovar Durban, secondly to describe the genomics analysis of this isolate and finally to discuss the possible source of infection.

## Case presentation

A 6-month-old patient was admitted in the pediatric emergency Department of CHC MontLégia Hospital (Liège, Belgium) with symptoms of fever and vomiting. The mother described a clinical picture with coughing and a runny nose. Medical history revealed that the patient, who was born in Belgium, had no previous serious infection and was vaccinated following the national scheme [7]. Two weeks prior to the emergency consultation, the patient was hospitalized in another pediatric hospital, also for fever and vomiting, which lead to intravenous rehydration for 72 h. During this time, a stool analysis came back positive for *S. enterica* serovar Durban so an oral antibiotic treatment was started with amoxicillin for seven days.

When the patient arrived at our hospital, he was admitted with a stable overall condition, exhibiting no fever and showing normal vital sign ranges.

The physical examination was normal and included no depressed anterior fontanelle tone and no altered state of consciousness. The preclinical explorations are detailed in Table 1.

**Table 1:** Paraclinical results at admission

PARACLINICAL		RESULTS	UNITS	RANGE
Hemogram	Hemoglobin	11,7	g/dL	10,4–12,5
	Hematocrit	34,7%	%	30,5–36,4
	Platelets	582	× 10^3^/mm^3^	185–399
	Leucocytes	24,180	× 10^3^/mm^3^	7,70–13,10
	Neutrophils	17,26	× 10^3^/mm^3^	2,50–6,4
	Lymphocytes	5,710	× 10^3^/mm^3^	2,30–5,50
Biochemistry	C reactive protein	178,4	mg/L	< 5
	Glycemia	131	mg/dL	60–100
	Urea	32,7	mg/dL	11–36
	Creatinine	0,32	mg/dL	0,20–0,40
	Glomerular Filtration Rate	87,2	mL/min/1,73 m^2^	> 60
	Sodium	140	mmol/L	139–146
	Potassium	4,2	mmol/L	4,1–5,3
	Chloride	103	mmol/L	100–111
Veinous Blood Gas		Normal		
Chest X Ray		Normal		
Abdominal Ultrasound		Normal		

Blood count, complete biochemical analysis, acute-phase proteins determination, and blood culture were taken. Laboratory findings showed increased inflammatory markers (platelets and CRP). Electrolytes, glucose, creatinine, liver enzymes, and bilirubin were within normal ranges.

Because there were increased inflammatory markers without any explanation, the patient was kept on a close clinical watch with monitoring.

During this time, his clinical state deteriorated with a decreased level of activity and the presence of meningeal signs such as neck stiffness. Due to this evolution, a lumbar puncture for cellular and microbiological study was quickly performed to exclude meningitis. The lumbar puncture results are shown in Table 2 and confirm the bacterial meningitis diagnosis.

**Table 2:** Cerebrospinal fluid analysis

Color			Trouble		
			**RESULTS**	**UNITS**	**RANGE**
Biochemistry	Proteins		2,349	g/L	0,100–0,450
	Glucose		< 4	mg/dL	60–80
	Lactic acid		10.880	mmol/L	1,1–2,8
Cell counts	Red cells counts		110	mm^3^	0–4
	White cells counts		6 406/mm3	mm^3^	0–5
	Neutrophils		76	%	
	Lymphocytes		9	%	
PCR Test for *Escherichia coli K1*, *Haemophilus influenzae*, *Listeria monocytogenes*, *Neisseria meningitis, Streptococcus agalactiae*, *Streptococcus pneumoniae*, *Human alphaherpesvirus 5, Enterovirus*, *Human alphaherpesvirus 1*, *Human alphaherpesvirus 2*, *Human alphaherpesvirus 6*, *Human parechovirus*, *Human alphaherpesvirus 3*, *Cryptococcus neoformans/gattii* [8]		Negative			
Microbial culture		Positive for *Salmonella* (D group)			

Due to the clinical deterioration, an empirical antibiotic treatment with intravenous cefotaxime was quickly initiated at a dose of 200 mg/kg/day and the patient was admitted in the pediatric intensive care unit. The blood cultures collected at admission yielded negative results.

Both CSF and stool cultures came back positive for *Salmonella* (group O9) after 24 h of incubation. The isolated strain was sent for classification and further analysis to the NRC for *Salmonella*.

The strain was typed as *S. enterica* serovar Durban and it showed sensitivity to all types of antibiotics tested (amoxicillin, azithromycin, cefotaxime, ceftazidime, ciprofloxacin, colistin, ertapeneme, gentamicin, meropenem and trimethoprim).

This type of serovar is rarely seen in Belgium (0 – 6 cases annually in the period 2014 – 2021) and in the EU/EEA (11–33 cases annually in the period 2014–2020) [9].

**Fig. 1:**
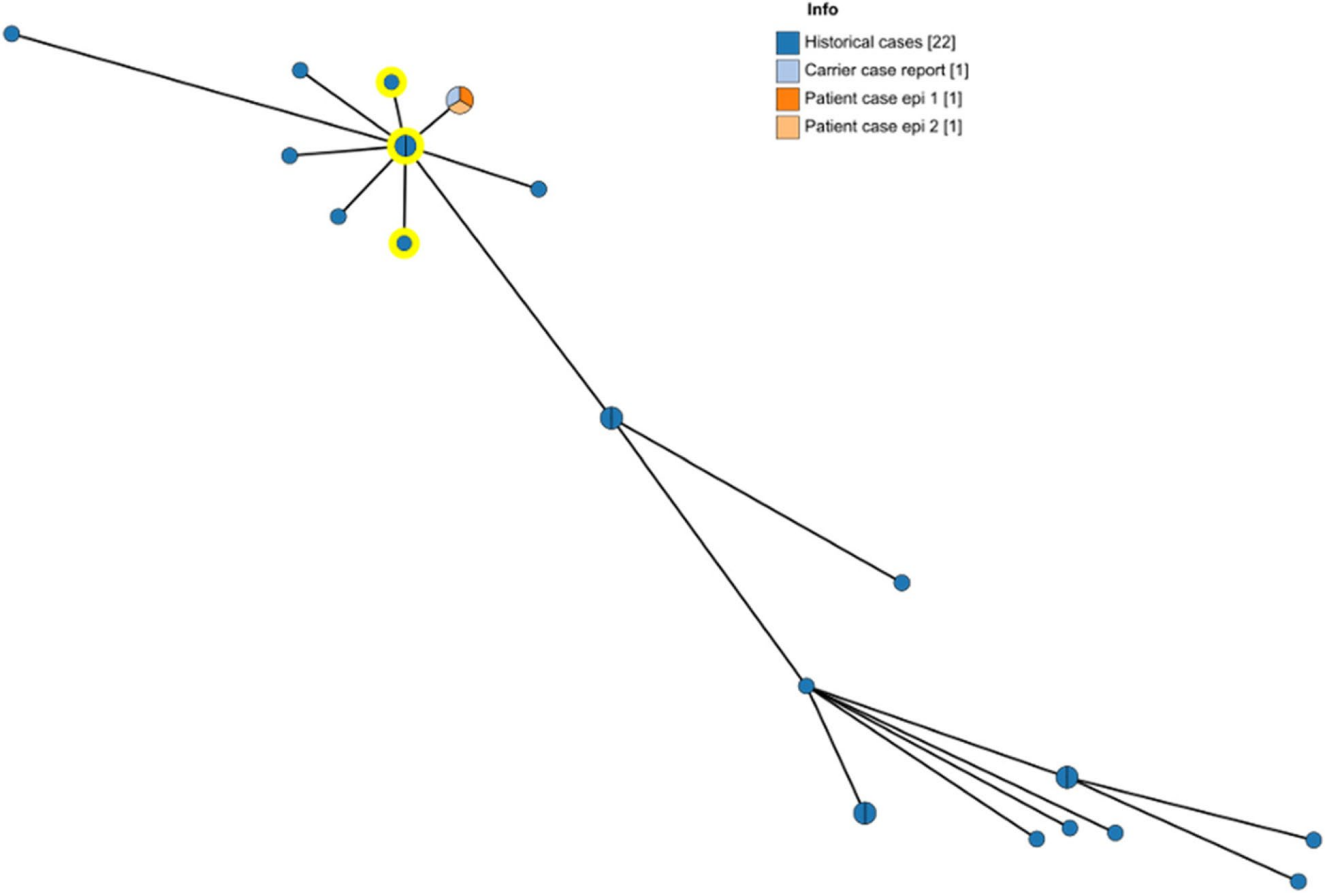
Minimum Spanning Tree using cgMLST data (EnteroBase scheme, https://enterobase.warwick.ac.uk/) of *S. enterica* serovar Durban of this case report and historical cases isolated from patients in Belgium between 2014 and 2021. Each node represents an isolate, with allelic differences indicated as branch length. Isolates with travel history to Guinea are highlighted in yellow. (epi = disease episode)

The CSF and stool isolateswere investigated for genetic relatedness by whole genome sequencing and confirmed to be genetically indistinguishable (0 allele differences, cgMLST scheme Enterobase, https://enterobase.warwick.ac.uk/). The screening of the genome of the isolate of the patient for antibiotic resistance markers confirmed the phenotypic results and indicated the isolate as susceptible for all known resistances [10, 11]. Further, genetic comparison with all other *S. enterica* serovar Durban cases isolated in Belgium during the period 2014 – 2021 (N = 22, Table 3) revealed a tight cluster with 8 other isolated cases (0 – 6 allele differences) (Fig. 1). Screening of the parents for *Salmonella* carriage resulted in a fecal sample of the father positive for *S. enterica* serovar Durban, which was also genetically indistinguishable (by cgMLST) from the case isolates.

**Table 3:** Historical *S. enterica* serovar Durban cases isolated in Belgium during the period 2014 – 2021. (UNK = unknown, Y = yes, N = no)

*Sample Id*	*Isolation year*	*Patient Age*	*Specimen*	*Clinical Info*	*Travel*	*Country*
*S14BD00872*	2014	1	Blood	Septicemia	Y	Guinea
*S14BD03902*	2014	UNK	Faeces	UNK	UNK	
*S15BD03255*	2015	11	Faeces	Gastroenteritis	N	
*S15BD04152*	2015	6	Faeces	Gastroenteritis	N	
*S15BD05605*	2015	19	Faeces	Gastroenteritis	N	
*S15BD10068*	2015	30	Faeces	UNK	N	
*S15BD10166*	2015	67	Faeces	UNK	N	
*S16BD05097*	2016	2	Blood	Gastroenteritis	N	
*S16BD06646*	2016	9	Faeces	Malaria + Gastroenteritis	Y	Guinea
*S17BD05858*	2017	2	Faeces	UNK	N	
*S17BD07615*	2017	0	Faeces	UNK	N	
*S18BD06055*	2018	1	Faeces	UNK	N	
*S18BD08552*	2018	24	Faeces	UNK	N	
*S19BD02073*	2019	2	Faeces	UNK	UNK	
*S19BD06302*	2019	1	Faeces	UNK	N	
*S19BD06559*	2019	1	Blood	cervical adenopathy	Y	Guinea
*S19BD06638*	2019	3	Faeces	UNK	UNK	
*S19BD08525*	2019	0	Faeces	bronchitis	UNK	
*S19BD09248*	2019	0	Faeces	UNK	UNK	
*S21BD04961*	2021	2	Faeces	UNK	Y	Guinea
*S21BD05077*	2021	5	Faeces	UNK	UNK	
*S21BD05152*	2021	57	Faeces	UNK	UNK	
*S22BD02061*	2022	41	Faeces	Carrier	N

Because of the very rare clinical presentation, an immunologic workup was done to rule out an immunodeficiency. Considering the patient’s age, the results were within the normal range (Table 4).

**Table 4:** Immunologic blood test

	**RESULTS**	**UNITS**	**RANGE**
CD3	58	%	51,8–74,2
	2802	/mm^3^	2284–4476
CD4	39	%	34,9–53,1
	1884	/mm^3^	1523–3472
CD8	17	%	12,8–27,1
	821	/mm^3^	524–1583
CD4/CD8 ratio	2,3	ratio	1,5–3,8
CD19	36	%	17–37,2
	1739	/mm^3^	776–2238
CD16 CD56	4	%	4–15,1
	193	/mm^3^	230–801
Total proteins	64,4	g/L	44–76
IgG	8,37	g/L	2,20–9
IgA	0,76	g/L	0,08–0,80
IgM	0,90	g/L	0,35–1,25
Complement C3	1,92	g/L	0,70–1,40
Complement C4	0,28	g/L	0,12–0,36
CH50	> 95	U/mL	41,7–95,1

**Fig. 2:**
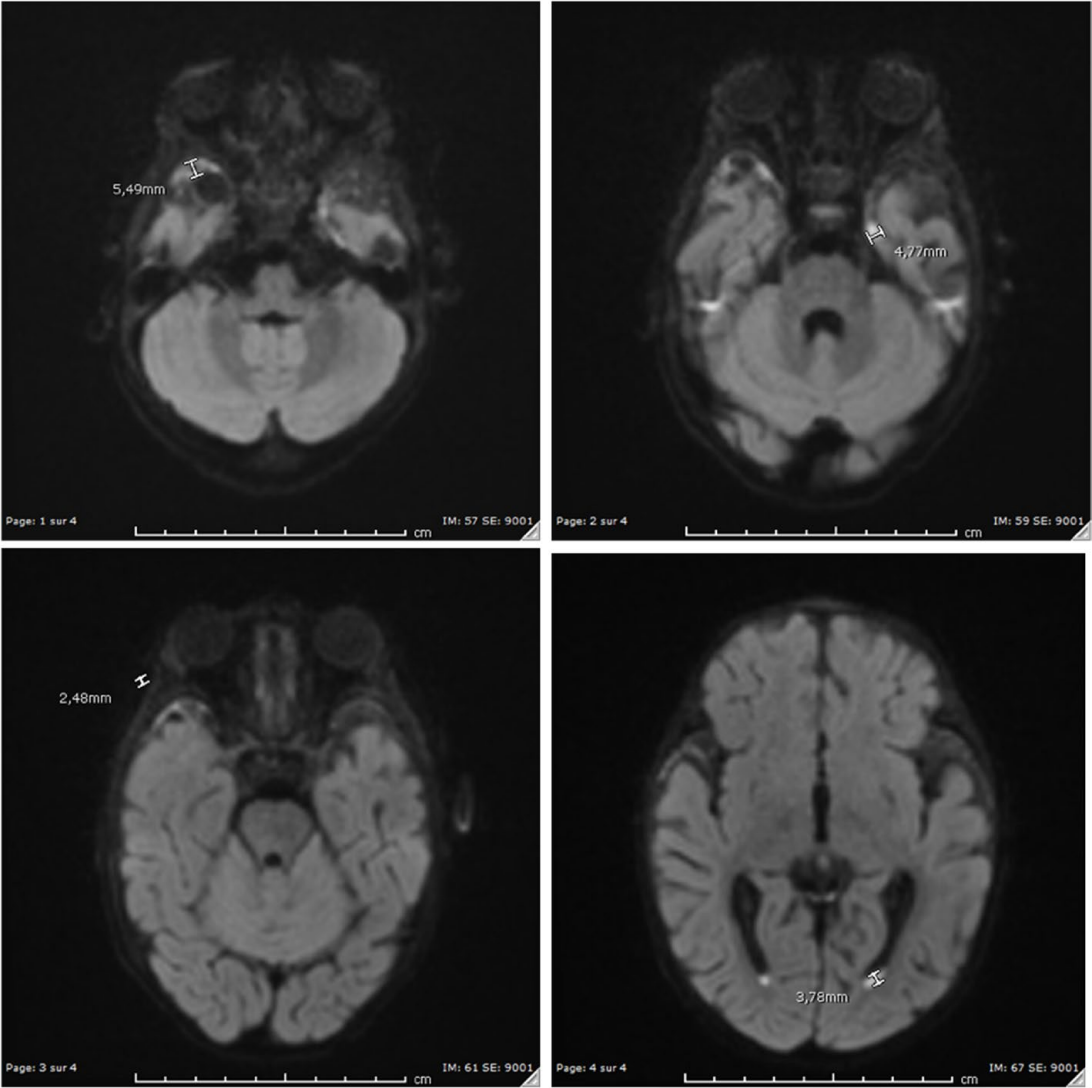
Brain magnetic resonance imaging showing purulent suffusion on day 2 during the first admission

A supplementary examination was conducted using an EEG, which revealed normal brain activity during wakefulness. Further exploration through an MRI showed a purulent suffusion in the lateral ventricles consistent with clinical finding of acute meningitis and pyogenic ventriculitis. The images showed no brain abscess or extra-axial empyema (Fig. 2). The microbiological monitoring was done with daily stool cultures which came back negative after 4 days of intravenous treatment. The clinical evolution of the patient throughout hospitalization was good and he was discharged after 28 days of intravenous antibiotic therapy through the use of cefotaxime. A month later, cerebral MRI showed a persistent frontal purulent suffusion of a diameter of 5 mm but still no brain abscess or extra-axial empyema. The patient was scheduled for follow-up MRI and regular visits to pediatric consultations to assess the neurological development of the patient. The follow up cerebral MRI showed a progressive reduction of the frontal purulent material. Till date, the patient shows no neurological sequelae.

## Discussion and conclusions

*Salmonella* targets the gastrointestinal tract and, for immunocompetent patients, is eliminated by the immune response. However, children have an immature immune system and are more likely to develop complications like meningitis as described in our case.

When a *Salmonella* infection is suspected or found, it is important to send the *Salmonella* isolates to the NRC so they can confirm the identification and serotype by agglutination tests. There are numerous *Salmonella* serotypes and the most frequently associated with meningitis are *Salmonella* serovar Typhimurium (75–88%), followed by *Salmonella* serovar Enteritidis (8–16%) and less frequently *Salmonella* serovar Typhi (1–4%) [12–14]. Other serovar very rarely cause meningitis.

The patient presented in this case first developed a rather mild episode of salmonellosis with gastrointestinal symptoms. This initially appeared to be successfully treated by antibiotics (amoxicillin), yet one month later, the patient had a relapse with a more severe clinical presentation of bacterial meningitis.

The isolates of both infectious episodes were further typed by the NRC and confirmed to be *S. enterica* serovar Durban and were genetically indistinguishable. Further genetic comparison with historical *S. enterica* serovar Durban cases isolated in Belgium revealed a tight cluster with 8 other isolates.

Epidemiological investigations revealed that no family members had a history of fever or diarrhea. The mother reported no pets or contact with farm animals. The family is originally from Guinea but have not travelled there for part two years, neither had contact with people who had recently travelled. It is interesting to note that 4/8 related historical Belgian cases of *S. enterica* serovar Durban reported recent travel to Guinea at their time of illness. It is also known that *Salmonella* can cause chronic infections which could persist for years, and although infected individuals are highly contagious, they are typically asymptomatic, making the identification of carriers very difficult. The situation is further complicated by the fact that approximately 1 out of 4 carriers experience no clinical manifestations during the acute phase of the disease [15]. Carrier identification is not automatically done for the reason that it could require multiple stool samples because of the intermittent shedding over a long period of time which would be difficult to achieve.

Finding a link with Guinea in our patient and in light of our investigations, a fecal culture was collected from his parents which turned out positive with a genetically indistinguishable *S. enterica* serovar Durban strain from the father. The parents being totally asymptomatic as previously stated, transmission from the healthy father as carrier seems therefore the most plausible route of infection.

We described a case of *Salmonella* infection in an immunocompetent patient living in an industrialized country. The infant developed meningitis as complication of systemic infection probably due to his young age.

According to our experience, an early diagnosis based on recognition of acute neurological signs and laboratory findings associated to a prompt and appropriate antibiotic therapy for at least four to six weeks can improve the outcome of the patient and reduce the risk of neurological sequelae.

In our case, further typing and WGS comparison of the strain hinted at the epidemiological link. We investigated the origin of the family which led to the confirmation of the carrier state of one of the parents. This highlights the added value of enhanced molecular surveillance to investigate the possible source of infection and might help in investigation of outbreaks.

## Data Availability

All relevant data are in the paper.
